# Reverse shoulder arthroplasty in complex fractures of the proximal humerus: results after 7 years of follow-up

**DOI:** 10.1186/s10195-021-00597-5

**Published:** 2021-09-24

**Authors:** Alberto Izquierdo-Fernández, Marta Gómez-Rodríguez, Maite Urbano-Luque, Manuel García-Carmona, Rafael Quevedo-Reinoso, José Carlos Minarro

**Affiliations:** 1 Orthopaedics and Traumatology Department, University Hospital Reina Sofía, Córdoba, Spain; 2 Calle Buenos Aires 5B, 14006 Córdoba, Spain

**Keywords:** Reverse shoulder arthroplasty, Proximal humerus fracture, Elderly patient, Outcomes, Scapular notching

## Abstract

**Background:**

There is still little information about the long-term results of clinical and radiological evolution in patients older than 65 years with complex proximal humerus fractures (CPHF) treated acutely with reverse shoulder arthroplasty (RSA). The aim of this paper was to evaluate function and results 7 years after surgery.

**Material and methods:**

A prospective cross-sectional cohort study was designed for this purpose. Patients who underwent RSA surgery during 2012 because of a CPHF were included. The surgical approach was randomized (deltopectoral vs anterosuperior). Functional activity, evolution of tuberosities and evidence of scapular notching 7 years after surgery were analyzed.

**Results:**

After evaluating 32 patients, the Constant score improved from 64.83 in the first year to 69.54 at 7 years postoperative. Results were independent of the approach used. Functional outcomes were poorer in patients with scapular notching and when tuberosities were resorbed or displaced.

**Conclusions:**

At 7 years, function in patients undergoing RSA after CPHF demonstrated improvement in all patients except those who developed scapular notching or when tuberosities did not consolidate in an anatomical position. These results are completely independent of the approach used.

**Level of evidence:**

III Controlled cohort study.

## Introduction

Reverse shoulder arthroplasty (RSA) was first developed as an alternative treatment for rotator cuff arthropathy. Since then, it has proven to be an effective implant for many shoulder disorders. For this reason, there has been a gradual increase in the last decade in its use for proximal humerus fractures in elderly patients [1]. Clinical studies of RSA in this kind of fracture have shown improved outcomes when compared with hemiarthroplasty [2, 3].

However, in the current literature, it is difficult to find long-term data about clinical and radiological results in patients over 65 years with complex fractures of the proximal humerus treated with RSA [4–6]. This may be due to many factors. The first of them might be that orthopedic surgeons are still developing in this field and there are no solid long-term results yet. But there are other factors that may influence these results, such as the approach used, the condition of the tuberosities, and the appearance of scapular notching.

The aim of this paper was to evaluate the medium-term functional activity of complex fractures of the proximal humerus treated by a reverse shoulder prosthesis. Moreover, clinical results are compared with the two main approaches used, the final state of the tuberosities and the development of scapular notching.

## Material and methods

A prospective cross-sectional cohort study was designed to include patients who were treated at our hospital during 2012 for a complex proximal humerus fracture and treated by reverse shoulder replacement. The cohort for this study was analyzed on two occasions: (1) 12 months postoperatively, and (2) after 7 years, to determine whether or not functional activity decreases with time.

The inclusion and exclusion criteria are shown in Table 1.

**Table 1:** Inclusion and exclusion criteria

Inclusion criteria	Exclusion criteria
Age > 65 years 3- or 4-part fractures Fracture-dislocation Fracture with comminution of tuberosities Fracture with prior rotator cuff disease Initial treatment with reverse prosthesis	Pathological fractures Refractures Open fractures Plexus injury Deltoid muscle disorders Severe cognitive impairment according to SPMSQ (short portable mental status questionnaire) Prior shoulder surgery

The surgery was always performed by the same surgeon and the implant used was the stem-cemented Delta XTEND reverse prosthesis system from DepuySynthes (DePuy Orthopaedics, Warsaw, IN, USA).

The choice of which approach to use was randomized according to the history number of the patient. If the number was even, a deltopectoral approach was used, and if the number was odd, an anterosuperior approach was used. All patients were assessed by the rehabilitation department following surgery.

Functional activity and quality of life were evaluated through the Constant score and the Hospital for Special Surgery (HSS) score, 1 and 7 years after surgery [7, 8]. All of these measures are expressed as mean ± SD (standard deviation).

Radiographic controls were performed by protocol at 3, 6, and 12 months and annually afterwards (true AP, axial and scapular plane projections). For this study, 12-month and 7-year X-rays were used to evaluate scapular notching, the loosening of the prosthetic components, and the condition of the tuberosities. The existence of notching was classified according to the classification of Nérot and Sirveaux [9] as grade 1: limited to the external pillar; grade 2: in contact with the inferior screw; grade 3: extension beyond the inferior screw; and grade 4: extension beyond the baseplate of the prosthesis. The condition of the tuberosities was also determined by X-ray and divided into three groups: normal (N) when the tuberosities consolidated in the normal anatomical location, resorption (R) when the tuberosities were absent, and displaced (D) when the tuberosities consolidated in a poor non-anatomical position [10, 11].

SPSS software (IBM Inc, Armonk, NY, USA) was used for the statistical analysis. A descriptive analysis of the variables studied was performed and Student’s *t-*test was used for independent samples (*p* < 0.05) to determine whether there were any improvements in terms of functionality (Constant and HSS scores). Pearson’s chi-square test was used to determine whether differences in functionality depended on the type of surgical approach. An analysis of variance test (ANOVA) was performed to determine whether the functional results depended on the state of the tuberosities or the appearance of scapular notching. This study was approved by the ethics committee of our hospital, reference number 279-3954 (Protocolo COTINV: ver 3.1).

## Results

In 2012, a total of 43 patients underwent reverse shoulder replacement for a complex fracture of the proximal humerus. Of them, 40 patients met the inclusion criteria (35 patients with 3- or 4-part fractures and 5 with fracture-dislocation). During the follow-up period, eight patients were excluded, four due to death and four who were lost to follow-up; therefore, 32 patients remained in the study cohort (3 men and 29 women). The mean age was 74.14 years (range 65–87). A deltopectoral approach was used in 13 patients (40.6%) and an anterosuperior approach was used in 19 patients (59.3%). The primary data can be seen in Tables 2 and 3.

**Table 2:** We can see how the function (measured according to Constant and HSS) improved in the 7-year assessment in both approaches, although there were no statistically significant differences between the results of both approaches (*p* < 0.05)

	Anterosuperior approach (*n* = 19)		Deltopectoral approach (*n* = 13)	
Function	1 year	7 years	1 year	7 years
Constant	67.69	73.31	61.45	65.09
HSS	62.85	68.15	58.27	60.09

**Table 3:** We can see how the function (measured according to Constant and HSS) improved at the 7-year assessment in patients without notching (*p* < 0.05)

	No notching (n = 24)		With notching (n = 8)	
Function	1 year	7 years	1 year	7 years
Constant	64	72.06	66.5	64.5
HSS	60.31	64.69	61.63	64

In contrast, in patients with notching, there were no statistically significant changes in function at 7 years

**Fig. 1:**
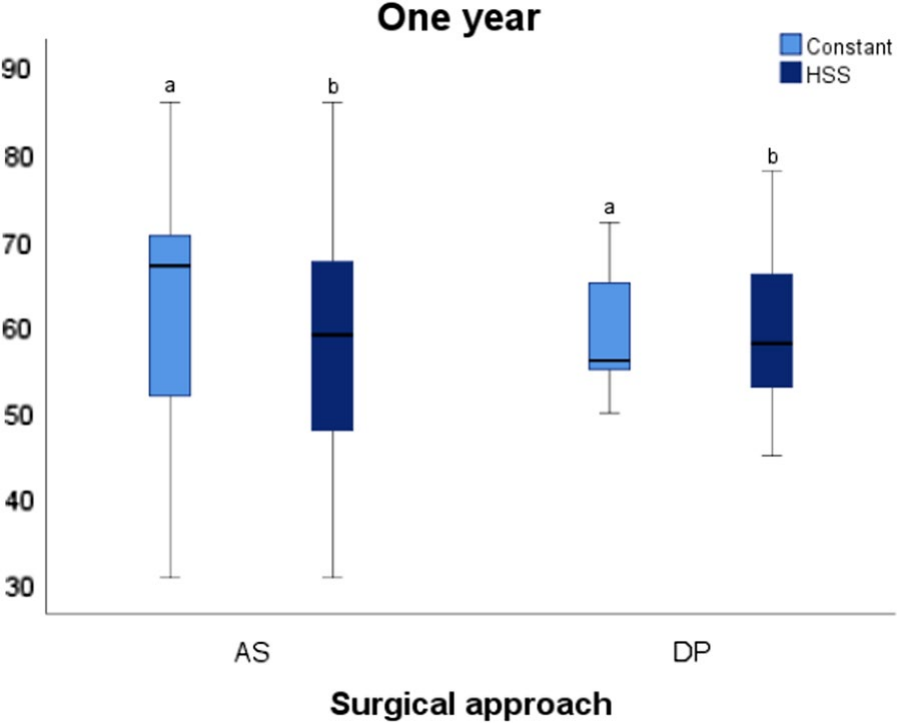
Boxplot charts where we can observe function (according to Constant and HSS) in both approaches used 1 year after surgery. *Same letters *indicate no statistically significant differences between them (*p* < 0.05, **a** and **b**). *AS* anterosuperior, *DP* deltopectoral

After analyzing the whole cohort the first year, the mean Constant was 64.83 (± 12.01) and the mean HSS was 60.75 (± 11.17). Evaluated independently, patients with an anterosuperior (AS) approach had a mean Constant of 67.69 (± 12.23) and a mean HSS of 62.85 (± 12.09). Patients with a deltopectoral (DP) approach had a mean Constant of 61.45 (± 11.37) and a mean HSS of 58.27 (± 9.95). With a paired Student’s *t-*test (*p* < 0.05), there were no statistically significant differences between the two approaches in terms of Constant and HSS (Fig. 1).

**Fig. 2:**
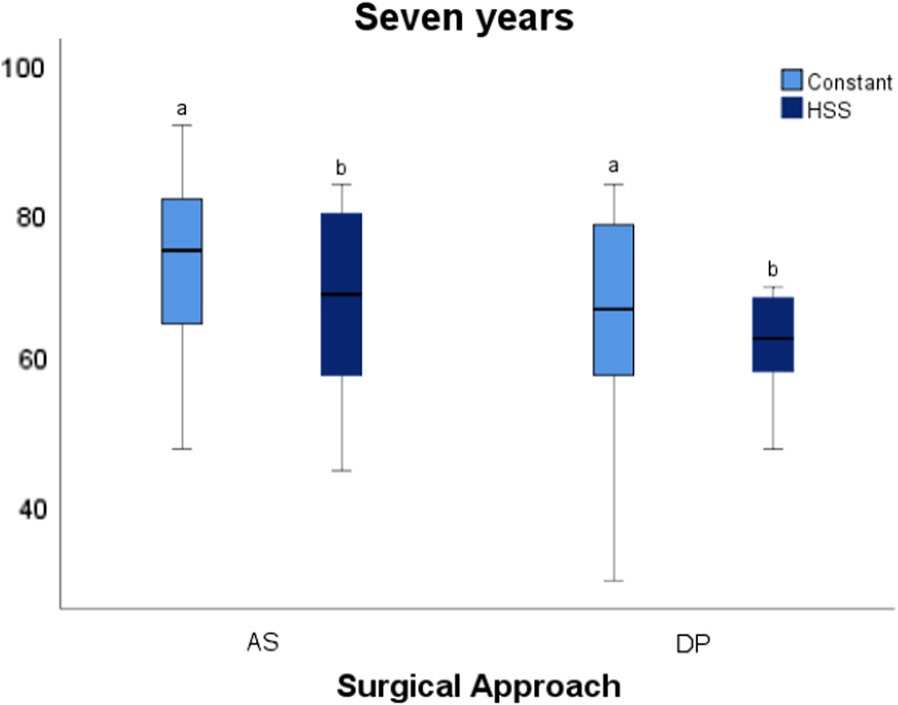
Boxplot chart where we can observe function (according to Constant and HSS) in both approaches used 7 years after surgery. *Same letters *indicate no statistically significant differences between them (*p* < 0.05, **a** and **b**). *AS* anterosuperior, *DP* deltopectoral

When the cohort was reviewed at 7 years, the mean Constant was 69.54 (± 14.82) and the mean HSS was 64.46 (± 12.78). Evaluated independently, patients with an anterosuperior (AS) approach had a mean Constant of 73.31 (± 13.22) and a mean HSS of 68.15 (± 12.74). Patients with a deltopectoral (DP) approach had a mean Constant of 65.09 (± 15.98) and a mean HSS of 60.07 (± 11.94). With a paired Student’s *t*-test (*p* < 0.05), there were no statistically significant differences between the two approaches in terms of Constant and HSS (Fig. 2).

In the radiographic assessment at 7 years, eight of the 32 patients had scapular notching (six grade 1 and two grade 2). Patients with no notching had a mean Constant of 64 (± 12.39) and a mean HSS of 60.31 (± 12.18) at 12 months and their function had improved significantly at 7 years (*p* < 0.05) to a mean Constant of 72.06 (± 12.89) and a mean HSS of 64.69 (± 11.77). On the other hand, patients who developed scapular notching went from a mean Constant of 66.5 (± 11.85) and a mean HSS of 61.63 (± 9.53) at 12 months to a mean Constant of 64.5 (± 17.96) and a mean HSS of 64 (± 15.49) at 7 years, with no statistically significant change. Among patients with notching, 3 underwent surgery by DP approach and 5 by AS approach, with no statistically significant differences between the approach and the existence of scapular notching.

**Fig. 3:**
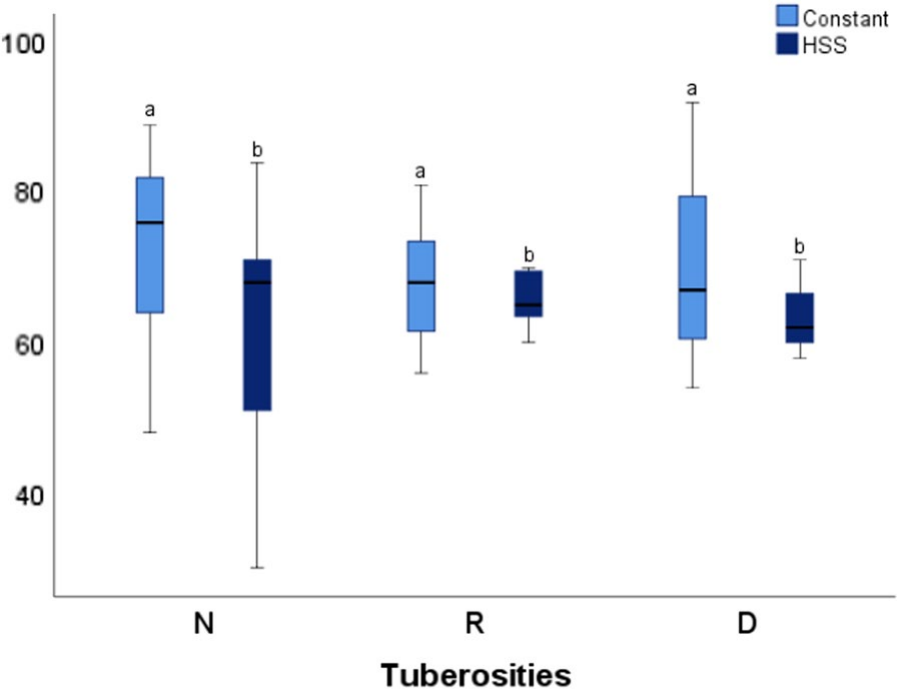
Boxplot chart where we can observe function (according to Constant and HSS) in relation to the state of tuberosities. *Same letters* indicate no statistically significant differences between them (*p* < 0.05, **a** and **b**). *N* normal, *R* resorbed, *D* displaced

When relating the functional results to the condition of the tuberosities, the mean Constant in the N group (22 patients) was 70.07 (± 17.08) and the mean HSS was 62.91 (± 15.64). The mean Constant in the R group (7 patients) was 67.85 (± 9), with a mean HSS of 67.85 (± 7.9). Finally, the mean Constant in the D group (3 patients) was 71 (± 19.31) with a mean HSS of 63.66 (± 6.65). There were no statistically significant differences (*p* > 0.05) with respect to the functional results and the condition of the tuberosities (Fig. 3).

During the follow-up period, the following major complications occurred: one periprosthetic fracture (treated with osteosynthesis) and one prosthetic dislocation (resolved through closed reduction with no need for replacement). No signs of component loosening were reported on follow-up control radiographs.

## Discussion

Fractures of the vertebrae, hip, distal radius, and humerus are the most common fractures in osteoporosis. The average risk of suffering a fracture of the humerus has been estimated as 12.9% for women and 4.1% for men over the course of their lives [12]. They represent the third most common fracture in individuals over 65 years of age [13, 14]. Our study shows how complex proximal humerus fractures (CPHF) in the elderly population can be treated by reverse shoulder arthroplasty, obtaining good results in the medium term.

Non-displaced and minimally displaced fractures are usually treated without surgery, with satisfactory outcomes [15–17]; meanwhile, displaced fractures, according to Neer’s criteria, usually require surgical intervention [18, 19]. Historically, complex fractures of the humerus include: four-part fractures, three-part fractures in osteoporotic bone, displacement fractures, and fractures that involve 40% to 50% of the joint surface [3].

**Fig. 4:**
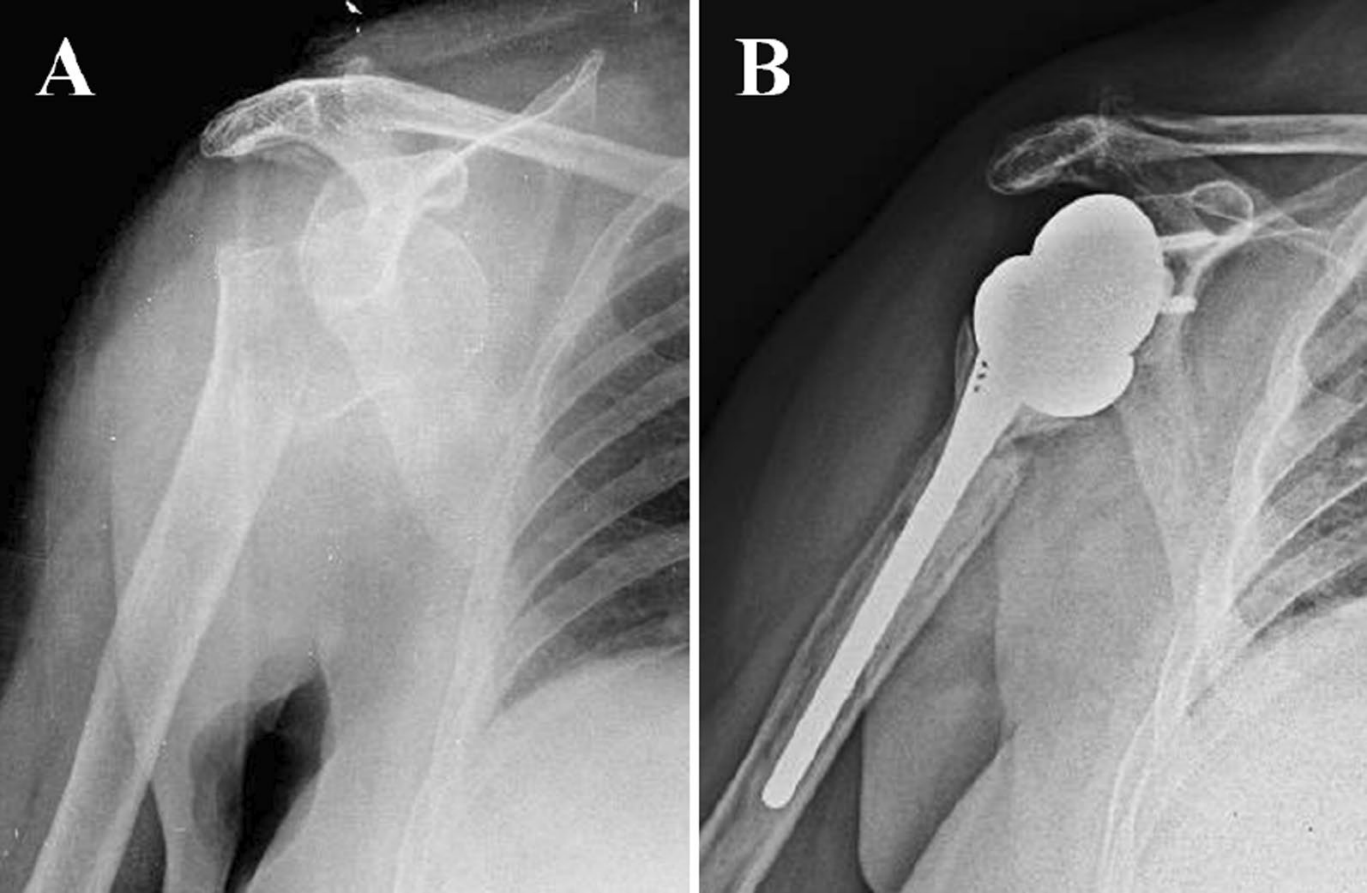
These images show X-rays of the same patient, with a complex dislocated fracture of the proximal humerus (**A**) and his follow-up after 7 years (**B**)

Since Grammont developed the reverse total shoulder arthroplasty (RSA) for cuff tear arthropathies in 1985 [20], its indications have increased, including its use for fractures of the proximal humerus in patients over 65 years old. Many studies recommend the use of RSA in fractures as an appropriate alternative [3, 6, 21–24]. This study is among the longest follow-up time in patients over 65 with reverse prosthesis due to trauma (complex fractures of the proximal humerus). We aimed to determine the relationship between functional results and important parameters such as the approach used, tuberosities, and scapular notching (Fig. 4).

The superior or anterosuperior (AS) approach was the original recommended approach for RSA by Grammont. As the procedure became more popular and indicated in more complex situations, many shoulder surgerons utilized the deltopectoral (DP) approach in an effort to gain better access to the proximal humerus [25, 26]. While some surgeons feel that the AS approach affords better and less invasive exposure to the glenoid surface, a controversy remains as to which is the best approach and which one to choose depending on the situation [25, 27].

Many authors have compared both approaches for rotator cuff arthropathy [25, 27–29]. All of them determined that both approaches have similar results. However, there are two complications that have been described with greater frequency in the AS approach: scapular notching and glenoid loosening [5, 6, 28, 30, 31]. In our study, we found no differences between scapular notching and the approach used and no glenoid loosening was reported, similar to other studies [32–34]. However, the number of patients is smaller than in rotator cuff arthropathy studies, so, in our opinion, it is not possible to draw conclusions based on the incidence of notch and the type of approach.

When relating functional outcomes and both the approaches used, results were similar, as reported previously in the literature [32–34]. Analyzing more carefully, although no statistically significant differences were found between the two approaches, there did seem to be a tendency for better clinical results with AS; future studies with a larger sample may clarify these results.

One of the greatest difficulties when implanting an RSA after a fracture involves fixation of the tuberosities. In fact, their proper consolidation has been described as one of the main prognostic factors for functional recovery [35]. Many studies show better functional outcomes after correct healing [10, 36–38]. In our study, 22 out of 32 patients (68.75%) had normal consolidation of the tuberosities, but this finding was not reflected in a difference of function compared with the rest of the patients (resorption and displaced tuberosities), as has already been described by other authors [6, 36].

Various authors argue that function in patients with an RSA decreases in time due to the micro-loosening of the components and depletion of the deltoid muscle [5, 10, 39]. However, as our study demonstrates, along with others [36, 37], functional results are not only maintained, but even improved over time (measured according to Constant and HSS scores). Moreover, scapular notching is a factor that is usually related to a significant decrease in function [32, 33, 40]. However, in our study, those patients who did not develop notching did improve their function over time, and patients with notching maintained their function at 7 years. The RSA has been shown to be an effective short- and medium-term treatment for a wide variety of shoulder conditions including complex proximal humerus fractures [34, 41–43]. The authors believe that the use of RSA instead of osteosynthesis may be an advantage in some patients, and that selected patients should include those with complex proximal humerus fractures over 65 years of age, with more than 3–4 displaced fragments, in which the tuberosities are highly comminuted or the osteosynthesis does not guarantee a good position for them. In such cases, the authors believe that results are more predictable and even better with RSA.

## Conclusions

Patients over 65 years old with a complex fracture of the proximal humerus who were treated with reverse shoulder arthroplasty, obtained very good results (constant and HSS scores) at 7 years of follow-up, demonstrating its effectiveness in the medium term. Moreover, those patients who did not develop scapular notching improved their function with respect to the first postoperative year. Neither the approach used for RSA implantation (deltopectoral or anterosuperior), nor the state of the tuberosities (normal, absent, or displaced) influenced the final functional outcomes.

## Data Availability

The datasets used and/or analyzed during the current study are available from the corresponding author on reasonable request.
